# Bridging neuromorphic computing and deep learning for next-generation neural data interpretation

**DOI:** 10.3389/fncom.2025.1737839

**Published:** 2026-01-08

**Authors:** Manyun Zhang, Tianlei Wang, Zhiyuan Zhu

**Affiliations:** 1 Key Laboratory of Songliao Aquatic Environment, Ministry of Education, Jilin Jianzhu University, Changchun, China; 2 College of Electronic Engineering, Southwest University, Chongqing, China; 3 Tianin Key Laboratory of Building Green Functional Materials, Tianjin Chengjian University, Tianjin, China

**Keywords:** brain–machine interface, computational neuroscience, deep learning, hybrid neural networks, neural decoding, neuromorphic computing

## 1 Introduction

The rapid advancement of electrophysiological techniques, brain imaging, and brain–machine interfaces (BMIs) has ushered neuroscience into an era of data explosion. Confronted with neural data that are high-dimensional, highly nonlinear, and exhibit complex temporal dependencies, conventional statistical and signal processing methods—often reliant on linear assumptions or low-dimensional projections—struggle to reveal the true mechanisms of brain activity (Livezey and Glaser, 2021). In response to this challenge, deep learning (DL) and neuromorphic computing (NC) have emerged as two promising yet conceptually distinct computational paradigms. Deep learning has demonstrated remarkable capabilities in data-driven modeling, achieving significant breakthroughs in neural signal decoding and cognitive state identification. However, its inherent limitations—high energy consumption, limited interpretability, and low biological plausibility—restrict its deeper application in computational neuroscience. In contrast, neuromorphic computing, inspired by the event-driven and local plasticity properties of biological neural systems, offers unique advantages in low-power adaptive processing. Nonetheless, it still faces challenges in training algorithms and scalability (Ivanov et al., 2022; Schuman et al., 2022). To address these complementary shortcomings, this article proposes a hybrid framework that integrates neuromorphic computing with deep learning, aiming to harmonize biological plausibility with high computational performance, thereby opening new pathways for developing next-generation models and tools for neural data interpretation (Liu et al., 2024).

## 2 Biological inspiration of neuromorphic computing

Neuromorphic computing seeks to emulate the structural and functional organization of the brain, enabling computational systems to operate in ways that resemble biological neural processing. Its central idea is to incorporate event-driven and asynchronous communication, allowing computation to occur only when an event is triggered rather than at fixed time intervals (Davies et al., 2021). This mechanism markedly reduces redundant operations and energy consumption, mirroring the physiological principle by which neurons fire only when their membrane potential surpasses a threshold.

At the heart of neuromorphic computing lies the Spiking Neural Network (SNN), which represents information through discrete electrical impulses, or spikes, that are temporally encoded to convey meaning (Guo et al., 2022; Michaelis et al., 2022). Learning in SNNs is commonly governed by Spike-Timing-Dependent Plasticity (STDP)—a local rule that adjusts synaptic strength based on the precise timing of pre- and postsynaptic spikes. This biologically grounded mechanism enables SNNs to capture causal relationships in neural activity, making them more faithful to real neural dynamics than conventional Artificial Neural Networks (ANNs). Consequently, SNNs excel in handling temporal sequences, sparse representations, and real-time responses.

In recent years, hardware implementations such as Intel Loihi, IBM TrueNorth, and BrainScaleS have demonstrated the promise of neuromorphic architectures for low-power, massively parallel computation (Davies et al., 2021; Michaelis et al., 2022). For example, the Loihi chip integrates on-chip plasticity circuits that support localized learning, achieving power efficiency several orders of magnitude better than traditional GPUs. Simultaneously, rapid advances in memristor technology have introduced new opportunities for neuromorphic hardware (Guo et al., 2022; Duan et al., 2024; Xiao et al., 2024). Memristors—devices that exhibit non-volatility, tunable conductance, and synapse-like behavior—enable in-memory computing, merging data storage and processing to emulate synaptic functionality directly on the chip.

Despite these advances, several challenges remain. The discrete nature of spikes makes training difficult, as standard backpropagation cannot be directly applied. Scalability also remains a concern: current systems struggle to maintain learning efficiency and robustness in large-scale data environments. Furthermore, the ecosystem of algorithms and software frameworks is still immature, lacking standardized interfaces for widespread adoption (Schuman et al., 2022). A recent Nature report highlights key breakthroughs in inter-chip communication, scalable architecture, and event-driven scheduling, signaling an important step toward large-scale, next-generation brain-inspired computing systems (Kudithipudi et al., 2025).

## 3 Breakthroughs and limitations of deep learning in neural data analysis

As the dominant paradigm in contemporary artificial intelligence, deep learning (DL) has achieved remarkable success in the analysis of neural data owing to its hierarchical feature extraction and nonlinear approximation capabilities (Livezey and Glaser, 2021; Zhou et al., 2024). Architectures such as Convolutional Neural Networks (CNNs), Recurrent Neural Networks (RNNs), and Transformers have been widely applied to the decoding of electroencephalography (EEG), local field potentials (LFP), and functional magnetic resonance imaging (fMRI) signals. These models can automatically extract multilayer representations, enabling the discovery of hidden relationships between neural activity and cognitive states.

In practical applications, CNNs have demonstrated high accuracy and robustness in EEG-based emotion recognition and brain–machine interface (BMI) command classification tasks (Li et al., 2024). RNNs and Long Short-Term Memory (LSTM) networks have exhibited outstanding capabilities in modeling the temporal dynamics of neural signals and predicting brain activity patterns (Bittar and Garner, 2022). Moreover, Transformer-based architectures have shown a superior ability to capture global dependencies across multimodal neural datasets, significantly improving the interpretability and scalability of neural decoding frameworks (Chen et al., 2023). Collectively, these advances indicate that deep learning not only facilitates complex pattern recognition in brain data but also provides statistical insights into the structure of neural processes.

However, the advantages of deep learning are accompanied by several intrinsic limitations. First, artificial neural networks rely on continuous activation functions and global backpropagation, which diverge from the local learning and synaptic plasticity observed in biological neural systems, resulting in limited biological plausibility. Second, the computational process of deep networks depends on large-scale matrix multiplications and parallel processing, leading to extremely high power consumption—orders of magnitude greater than that of biological brains. Third, deep models often suffer from poor interpretability; although their predictive performance is high, the internal representations rarely align with specific neurophysiological structures or mechanisms. In addition, conventional deep learning models lack temporal precision, making it difficult to accurately capture spike-based neural dynamics with millisecond resolution (Klos and Memmesheimer, 2025; Guo et al., 2023).

Therefore, while deep learning has proven powerful in feature extraction and cognitive modeling of neural data, achieving a genuine transition from “prediction” to “understanding” requires the incorporation of computational paradigms that align more closely with neurophysiological mechanisms. Neuromorphic computing offers a biologically inspired and energy-efficient complement to deep learning, paving the way toward a more interpretable and power-efficient framework for neural data modeling.

## 4 A hybrid framework integrating neuromorphic computing and deep learning

**Figure 1:**
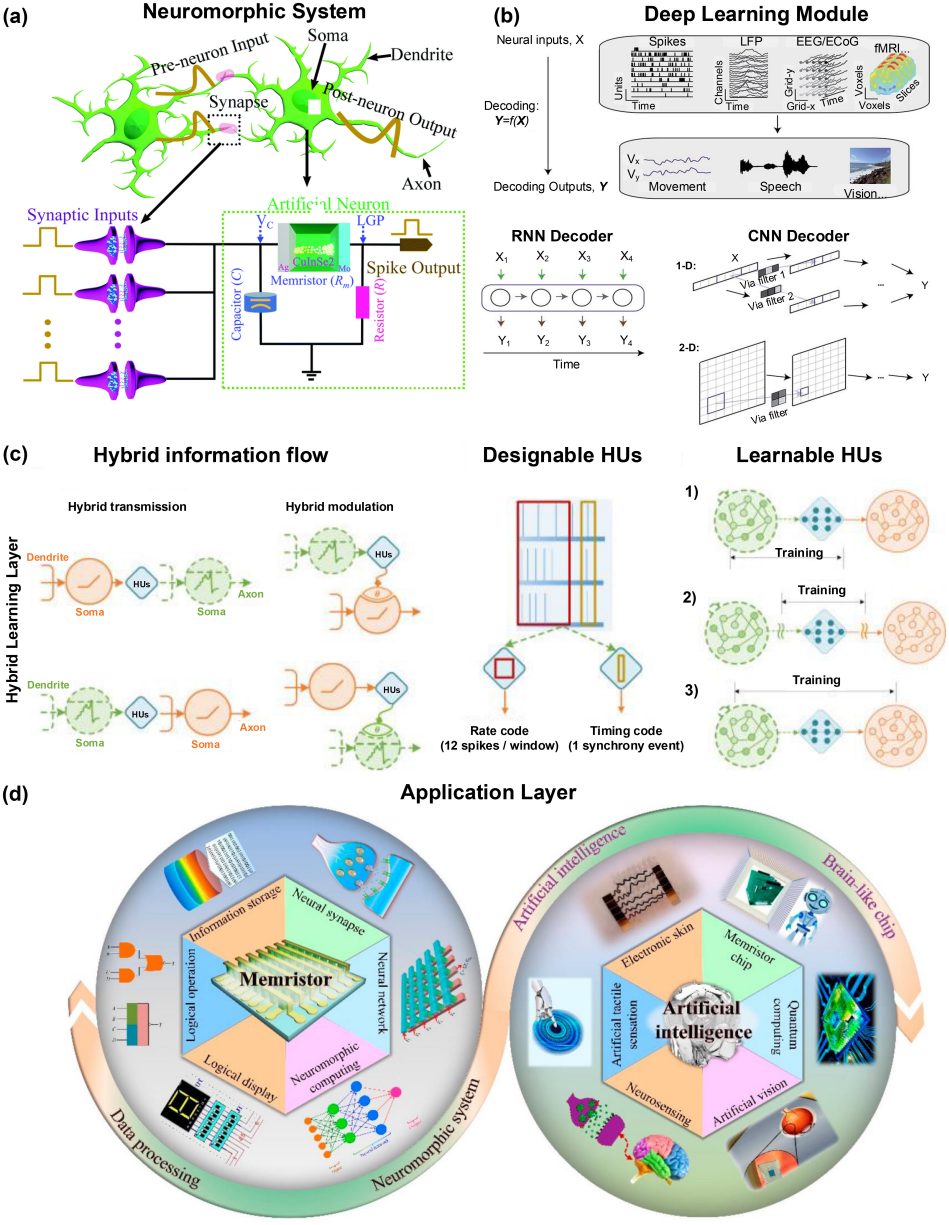
Conceptual framework of integrating neuromorphic computing and deep learning for neural data interpretation. **(a)** Neuromorphic system schematic showing the correspondence between biological neurons and memristor-based artificial synapses, illustrating spiking behavior and spike-timing-dependent plasticity (STDP). **(b)** Hybrid learning layer demonstrating the integration of spiking neural networks (SNNs) and artificial neural networks (ANNs) into a unified hybrid architecture for event-to-vector transformation. **(c)** Deep learning module showing representative deep architectures, including Transformer, RNN, and CNN, applied to neural decoding and cognitive modeling. **(d)** Application layer depicting memristor-based artificial intelligence chips and their potential uses in neural signal analysis, brain–machine interfaces, and cognitive modeling. **(a)** Adapted with permission from Guo et al. (2022). Copyright 2022, Royal Society of Chemistry. **(b)** Reprinted with permission from Livezey and Glaser (2021). Copyright 2021, Oxford University Press. **(c)** Reproduced with permission from Zhao et al. (2022). Copyright 2022, Nature Publishing Group. **(d)** Reproduced in part with permission under a Creative Commons License from Sun et al. (2023). Copyright 2023, American Chemical Society.

To reconcile the strong representational capacity of deep learning with the biological plausibility of neuromorphic computing, this work proposes a Hybrid Neuromorphic–Deep Learning Framework (Liu et al., 2024; Zhao et al., 2022). Figure 1 illustrates the overall conceptual framework of this hybrid approach. The framework integrates event-driven spiking computation with end-to-end deep feature learning, forming a multilayer neural data-processing system that is interpretable, energy-efficient, and aligned with neurophysiological dynamics.

At the architectural level, the framework consists of three major components: a neuromorphic front-end, a hybrid learning layer, and a deep interpretation back-end. The neuromorphic front-end extracts event-driven signals—such as spike trains—from multimodal neural recordings while performing noise suppression and initial temporal encoding (Jin et al., 2023). Through the use of spiking neural networks (SNNs) or memristor-based neuromorphic circuits, this stage enables low-power, real-time preprocessing at the hardware level (Duan et al., 2024; Jin et al., 2023). Recent progress in two-dimensional-material neuromorphic chips has further enhanced efficiency, sensitivity, and scalability, offering robust hardware support for hybrid neural architectures (Zhang et al., 2025).

The hybrid learning layer serves as the interface between the neuromorphic and deep-learning modules. Its role is to map sparse spike-based events into higher-level feature vectors. This can be achieved using surrogate-gradient optimization or biologically inspired local plasticity rules (Zhou et al., 2024; Chen et al., 2023), integrating local learning mechanisms with global gradient-based adaptation for improved interpretability and flexibility.

To operationalize the event-to-vector transformation within this layer, several established spike-to-vector conversion and differentiable training strategies can be adopted. For example, (Meng et al. 2022) introduced a differentiable spike-representation learning method that maps temporal spike sequences into continuous vector spaces, enabling cross-domain transformation from event-based signals to feature embeddings. Similarly, (Zhang et al. 2023) demonstrated that surrogate-gradient–based direct training in hybrid SNN–ANN networks can effectively extract and vectorize event-driven features. Furthermore, hybrid neural frameworks such as the Hybrid Neural Network (HNN) proposed by (Zhao et al. 2022) have verified that constructing learnable interaction layers between ANNs and SNNs enables efficient cross-domain feature projection, providing a feasible technical route for implementing the hybrid module in our framework. In addition, the analysis by (Liu et al. 2024) on the mechanisms and information flow in hybrid neural systems offers further theoretical support for the mapping strategy adopted here.

The deep interpretation back-end applies advanced neural models—such as Transformers, graph convolutional networks (GCNs), or recurrent neural networks (RNNs)—for pattern recognition, brain-state decoding, and cognitive representation learning. These stage further benefits from pre-training and knowledge-transfer mechanisms, improving generalization and abstraction quality (Liu et al., 2024; Zhao et al., 2022). Information flows from event-driven spike streams at the neuromorphic front-end, through the hybrid mapping layer, to high-level cognitive inference in the deep model. With a closed-loop design, feedback from the deep model can dynamically regulate neuromorphic parameters, enabling online learning and forming an adaptive, self-optimizing neural system.

Compared with the HNN framework introduced by (Zhao et al. 2022), which primarily targets efficient heterogeneous ANN–SNN co-inference, the proposed framework extends beyond coupling mechanisms to incorporate a neuromorphic sensory front-end and a deep interpretive back-end. This results in a cross-scale architecture spanning event encoding, hybrid feature learning, and neurodynamics-aligned interpretation. Consequently, the present framework emphasizes event-driven representation, biological interpretability, and alignment with neurophysiological mechanisms, offering broader conceptual scope and greater relevance for neural data analysis.

This hybrid framework offers several notable advantages. Event-driven computation reduces redundant operations and energy consumption. Spike-based representations correspond directly to neuronal firing patterns, improving interpretability. Deep neural models provide scalable abstraction for high-dimensional neural datasets. Finally, the integration of local plasticity with online adaptation imparts robustness and flexibility, forming a promising foundation for next-generation self-adaptive brain-machine systems.

## 5 Discussion and outlook

The integration of neuromorphic computing and deep learning represents not only a technological complementarity but also a profound paradigm shift in computational neuroscience (Liu et al., 2024; Meng et al., 2022). This cross-level integration provides a systematic pathway for neural information processing that bridges biological inspiration and high-dimensional modeling, allowing models to simultaneously capture neurodynamic plausibility and the abstract representational power of deep networks. Through this hybrid framework, computational neuroscience is gradually transitioning from mere signal fitting toward functional interpretation of neural mechanisms.

In the domain of neural encoding and decoding, hybrid models can more accurately characterize the dynamic firing patterns of neuronal populations, enabling precise recognition of complex neural processes such as motor intention, perceptual representation, and cognitive load (Livezey and Glaser, 2021). By combining the temporal precision of spike-based events with the hierarchical representations of deep networks, researchers can achieve a multi-scale description of neural information, uncovering the diversity and plasticity inherent in neural coding.

For brain connectivity modeling, integrating spike-driven event models with Graph Neural Networks (GNNs) offers a promising approach to uncover causal interactions and functional topologies among neural circuits (Zhao et al., 2022). Such frameworks not only facilitate the reconstruction of dynamic brain networks but also provide computational insights for the early diagnosis and intervention of neurological disorders such as epilepsy and Alzheimer's disease.

In brain–machine interfaces (BMIs) and neural rehabilitation, the hybrid architecture enables real-time signal decoding on low-power neuromorphic hardware, supporting adaptive and online-learning-based neural communication systems (Duan et al., 2024; Sun et al., 2023). By combining the efficient temporal processing of spiking neural networks with the high-level pattern recognition capability of deep learning, these systems can dynamically adjust decoding strategies while maintaining energy efficiency—thereby enhancing self-learning capabilities for intelligent neuroprosthetic control and rehabilitation.

From a hardware–intelligence co-design perspective, the rapid progress of memristor technology and three-dimensional integrated circuits is driving a deep convergence between neuromorphic chips and AI accelerators (Xiao et al., 2024; Jin et al., 2023). This cross-layer collaboration enables brain-inspired learning and adaptive cognition in edge-computing environments, making it feasible to achieve efficient, real-time neural computation directly on localized devices.

Looking forward, the core objective of computational neuroscience will revolve around achieving a balanced trade-off between energy efficiency, interpretability, and scalability. By combining the biological realism of neuromorphic computing with the abstraction capabilities of deep learning, a new generation of neural intelligent systems may emerge—systems that not only elucidate the computational principles and information flow of the brain but also drive the advancement of adaptive intelligent chips, brain-inspired computing platforms, and next-generation brain–machine interface technologies.

Despite the significant potential of the hybrid neuromorphic–deep learning framework, several limitations remain that must be addressed in future research. First, event-driven encoding is inherently sensitive to noise and may not be suitable for neural recording modalities with low temporal resolution or high measurement noise—such as calcium imaging or fMRI—which restricts its applicability across modalities. Second, training hybrid ANN–SNN systems often incurs substantial computational overhead. Cross-domain gradient propagation can introduce instability, and the field still lacks a unified strategy for multimodal fusion across spike-based and continuous representations. Third, current neuromorphic hardware faces practical challenges, including variability and limited reproducibility in memristive devices, as well as bandwidth constraints in inter-chip communication. These factors hinder large-scale deployment and stable on-chip training. Finally, although the deep interpretation module enables powerful high-dimensional feature abstraction, its biological interpretability remains imperfect and cannot yet fully align with real neurophysiological mechanisms. Therefore, applying this framework to real neural data analysis and brain–machine interface systems will require careful balancing among algorithmic design, hardware implementation, and neuroscientific validation.

## 6 Conclusion

We argue that the fusion of neuromorphic computing and deep learning constitutes a paradigm shift for computational neuroscience, moving the field beyond isolated algorithms toward a holistic paradigm that embraces cross-level abstraction, biological plausibility, and stringent energy constraints (Liu et al., 2024). The synergistic integration of event-driven processing with deep hierarchical learning is pivotal, enabling not only a more profound interpretation of neural dynamics but also the co-design of intelligent and energy-efficient hardware. This hybrid framework establishes a new foundation for future research, poised to significantly advance our capabilities in decoding neural computation, diagnosing neurological disorders, and engineering adaptive, brain-inspired intelligence (Zhao et al., 2022; Sun et al., 2023).
